# Tannic Acid and Ethacridine Lactate Attenuate Markers of Stress-Induced Intestinal Barrier Dysfunctions in Murine Small Intestinal Organoids

**DOI:** 10.3390/biom15050650

**Published:** 2025-04-30

**Authors:** Louisa Filipe Rosa, Steffen Gonda, Nadine Roese, Stephan C. Bischoff

**Affiliations:** 1 Institute of Nutritional Medicine, University of Hohenheim, Fruwirthstr. 12, 70599 Stuttgart, Germany; louisa.homberg@uni-hohenheim.de; 2 MEDICE Arzneimittel Pütter GmbH & Co. KG, Kuhloweg 37, 58638 Iserlohn, Germany

**Keywords:** tannic acid, ethacridine lactate, gastrointestinal barrier, tight junctions, antimicrobial peptides, diarrhea, gastroenteritis, mucosal protection, inflammation, organoids

## Abstract

(1) Background: Tannacomp^®^ is a drug consisting of tannin albuminate, a complex of tannic acid (TA) and ethacridine lactate (Eta) used for treating acute and traveler’s diarrhea. TA is thought to modulate gastrointestinal barrier function, but the underlying mechanisms and whether Eta has similar effects remains unclear. (2) Methods: to investigate the effects of TA and Eta on the intestinal barrier, stress responses were induced in murine intestinal organoids by lipopolysaccharide (LPS) exposure or withdrawal of growth factors from cell culture medium (GF_Red_). Further, organoids were exposed to either TA (0.01 mg/mL) or Eta (0.002 mg/mL) and markers of inflammatory response and gut barrier function were assessed. (3) Results: TA and Eta reduced several inflammatory markers such as interleukin 6, interleukin 1β, tumor necrosis factor α, and myeloid differentiation primary response 88 in stressed organoids. In addition, TA and Eta attenuated LPS- and GF_Red_-mediated gut barrier dysfunctions, with normalization of tight junction, adherent junction and mucin gene expression and reduction of *Nod2*- and matrix metalloproteinase 7-dependent activation of antimicrobial peptides. (4) Conclusions: our data show that TA and Eta modulate markers of inflammation and the intestinal barrier and suggest novel mechanisms of action of this drug that could broaden its treatment indications.

## 1. Introduction

The combination of tannins and ethacridine lactate has been used for decades to prevent and treat acute, non-specific and traveler’s diarrhea [1,2,3]. Tannins are polyphenolic compounds which exert effects by precipitating proteins [4,5]. This so-called “astringent effect” leads to reduced absorption of toxic substances, thereby protecting the intestinal mucosa against further stress factors [1]. In addition, TA provides anti-inflammatory and antiproliferative activities [6]. Eta exhibits antiseptic–bacteriostatic effects against different pathogenic bacterial strains, such as *Staphylococcus aureus*, *Escherichia coli*, various *Salmonella* strains and *Shigella* [7]. Further, Eta improves symptoms such as abdominal cramps and increased intestinal passage through spasmolytic effects [1]. Recent evidence also points towards the fact that tannins and Eta exhibit therapeutic activity by modulating gastrointestinal (GI) barrier function [8]. Intestinal barrier dysfunctions, especially disturbances in tight junction (TJ) and adherent junction (AJ) protein formation, have become an important factor in the pathogenesis of several diseases such as GI infections and inflammatory bowel diseases (IBD) such as Crohn’s disease (CD), recurrent diarrhea [9] or intestinal metaplasia [10,11]. We and others have shown that decreased occludin (*Ocln*) and zonula occludens 1 (*ZO-1*) expression results in impaired intestinal permeability, promoting bacterial translocation and low-grade inflammation [12,13,14,15,16]. Further, reduced claudin (*Cldn*) 7 formation contributes to increased GI inflammatory processes and cell degeneration [17].

Another important defense strategy of the GI barrier is the formation and secretion of antimicrobial peptides (AMPs), which exhibit broad antibacterial, antiviral and antifungal effects, thereby protecting against pathogens and regulating the intestinal microbiota [18,19,20]. Mammalian AMPs can be classified into cathelicidins, regenerating gene 3 proteins and defensins [20,21]. Defensins can be divided into α-, β-, and δ-defensins [19]. While α-defensins are mainly produced by Paneth cells in the small intestine, β-defensins occur on epithelial surfaces, such as in colon or liver [22]. A compromised GI antimicrobial peptide defense has been found to increase the risk of several GI diseases such as IBD or enterocolitis [23,24]. For example, CD patients exhibited reduced ileal α-defensin expression [25,26], which was associated with defects of AMP regulators, such as NOD2 [27,28], or toll-like receptors (TLR) [29]. Further, reduced intestinal α-defensin expression led to disturbed intestinal microbiota composition and bacterial overgrowth, as well as decreased GI barrier integrity, promoting bacterial translocation [13,30]. However, the underlying mechanisms of impaired AMP defense in the context of GI diseases are still not fully understood. Besides a decrease in Paneth cell number and function, impaired signal-mediated induction and reduced proteolytic activation have been discussed [24,31,32].

There is evidence that TA exerts therapeutic effects by regulating GI barrier function. Oral gavage of TA in an oxidative stress mouse model resulted in an improved intestinal morphology and intestinal barrier by reducing *Cldn* and inducing *ZO-1* expression [8]. Additionally, TA treatment improved lactose-induced diarrhea in rats by ameliorating GI barrier integrity, thereby reducing serum zonulin levels and inducing ileal ZO-1 protein expression [33]. Further, human studies revealed that enrichment of food with tannin extracts modulated the intestinal microbiota, specifically by increasing short chain fatty acid (SCFA)-producing bacteria, such as members of *Lachnospiraceae* and *Ruminococcaceae* families [34]. However, there is little knowledge available about how TA or Eta regulate GI barrier function, especially in the context of inflammatory processes. In the present study, we investigated the effects of Tannacomp^®^, especially of the components TA and Eta, on GI barrier function and inflammatory responses during stress exposure. For this purpose, an in vitro murine organoid model was used, offering the possibility to generate in vitro three-dimensional cell structures, resulting in in vivo-like organ complexity. Stress was induced by adding proinflammatory LPS or by the withdrawal of growth factors from the cell culture medium. Organoids are a well-established tool to study mucosal immune responses and intestinal barrier function related to chronic GI diseases.

## 2. Materials and Methods

### 2.1. Organoid Cell Culture

#### 2.1.1. Isolation and Cultivation

In order to generate small intestinal organoids, C57BL6J mice were anesthetized using CO_2_ and crypts were isolated from the small intestine by using crypt isolation buffer (CIB, PBSO containing 0.5 M EDTA). A total of 500 isolated crypts were plated with 25 µL Matrigel (Corning B.v., Amsterdam, The Netherlands) and 300 µL crypt culture medium (CCM) was added, consisting of advanced DMEM/F12 (ThermoFisher Scientific, Karlsruhe, Germany) supplemented with 2 mM GlutaMax^TM^ (ThermoFisher Scientific, Waltham, MA, USA), 10 mM Hepes (Merck, Darmstadt, Germany), 100 ng/µL Noggin (PeproTech, East Windsor, NJ, USA), 1 µg/mL R-Spondin (PeproTech, East Windsor, NJ, USA), B-27™ supplement 1× (Invitrogen, Carlsbad, CA, USA), 1 mM N-Acetylcysteine (Sigma-Aldrich, Schnelldorf, Germany), 0.1 mg/mL Primocin (Invitrogen, Carlsbad, CA, USA) and 50 ng/mL EGF (Immunotools, Friesoythe, Germany). The resulting organoids were cultured for a minimum of seven days, as previously described [35].

#### 2.1.2. Medium Change and Splitting

Organoid growth was monitored by light microscopy and cell culture medium was changed every third day. For cell splitting, CCM was replaced with 500 µL wash buffer (advanced DMEM/F12) containing Pen (100 U/mL)/Strep (100 µg/mL) (ThermoFisher Scientific, Karlsruhe, Germany) and 7.5% BSA (solved in PBSO; Carl Roth GmbH, Karlsruhe, Germany). Organoids were mechanically disrupted followed by centrifugation at 200× *g* for 5 min, then washed repeatedly with 2 mL wash buffer and centrifuged at 200× *g* for 5 min. Cell pellets were suspended with 25 µL Matrigel, plated in a 48-well plate and 300 µL CCM was added.

### 2.2. Determination of Tannic Acid (TA) and Ethacridine Lactate (Eta) Concentration

For dose determination, the effects of TA and Eta on cell number and cell viability were investigated by using an MTT reduction assay. In addition, markers of intestinal barrier inflammation were analyzed by RT-PCR. For MTT assay, organoids were exposed to 6 µL TA (solved in PBSO, stock solution 50 mg/mL for final concentration of 1 mg/mL), or 6 µL TA (solved in PBSO, stock solution 5 mg/mL for final concentration of 0.1 mg/mL), or 0.6 µL TA (solved in PBSO, stock solution 5 mg/mL for final concentration of 0.01 mg/mL), or 6 µL Eta (solved in PBSO, stock solution 50 mg/mL for final concentration of 1 mg/mL), or 6 µL Eta (solved in PBSO, stock solution 5 mg/mL for final concentration of 0.1 mg/mL), or 0.12 µL Eta (solved in PBSO, stock solution 5 mg/mL for final concentration of 0.002 mg/mL), or with an equivalent amount of PBSO as control for 30 h. Then, 7 µL MTT solution (500 mg/mL, solved in PBSO) was added and organoids were incubated for 1 h at 37 °C. Supernatant was discarded, and organoids were incubated for 1 h at 37 °C with 20 µL SDS. Subsequently, 100 µL DMSO was added (1 h at 37 °C) and optical density was measured at 562 nm. Further, based on MTT assay measurements, ED50 and ED100 were calculated by using the following equation:

$$y=\frac{{OD}_{min}+({OD}_{max}-{OD}_{min})}{1+{\left(\frac{x}{ED50}\right)}^{h}}$$

### 2.3. Establishing Methods for Organoid Stress

Different stress conditions were tested in organoids by conducting the following experimental protocols: (i) reduction of growth factors R-Spondin and noggin in CCM, (ii) LPS exposure (100 µg/mL, 50 µg/mL, solved in PBS). For this purpose, R-Spondin and noggin, both activators of Wnt signaling pathway, were reduced (GF_Red_, Table 1) and organoids were stimulated with 300 µL GF_Red_, consisting of DMEM/F12 with 0.5 µg/mL R-Spondin, 50 ng/µL noggin, 1x B-27™ supplement, 1 mM N-Acetylcysteine, 0.1 mg/mL Primocin and 50 ng/mL mEGF. For the second protocol, organoids were stimulated with 15 µL LPS (solved in PBSO, stock solution 2 mg/mL for final concentration of 100 µg/mL), or 7.5 µL LPS (solved in PBSO, stock solution 2 mg/mL for final concentration of 50 µg/mL), or with a corresponding amount of PBS as control for 30 h. Total RNA was extracted, reverse-transcribed and relative gene expression of *Cldn7*, *Ocln*, interleukin (*IL*)-6 and *IL-1β* was calculated by comparison to housekeeping gene β-Actin by using the ΔΔ-Ct method (oligonucleotide primer sequences are listed in Table S1).

### 2.4. Exposure to Pharmacologic Molecules

To evaluate the effects of 0.01 mg/mL TA or 0.002 mg/mL Eta on GI barrier function and inflammatory processes, organoids were exposed to 0.6 µL TA (solved in PBSO, stock solution 5 mg/mL for final concentration of 0.01 mg/mL), or 0.12 µL Eta (solved in PBSO, stock solution 5 mg/mL for final concentration of 0.002 mg/mL, solved in PBSO), or GF_Red_ ± 0.6 µL TA (solved in PBSO, stock solution 5 mg/mL for final concentration of 0.01 mg/mL), or GF_Red_ ± 0.12 µL Eta (solved in PBSO, stock solution 5 mg/mL for final concentration of 0.002 mg/mL), or 7.5 µL LPS (solved in PBSO, stock solution 2 mg/mL for final concentration of 50 µg/mL) ± 0.6 µL TA (solved in PBSO, stock solution 5 mg/mL for final concentration of 0.01 mg/mL), or 7.5 µL LPS (solved in PBSO, stock solution 2 mg/mL for final concentration of 50 µg/mL) ± 0.12 µL ETA (solved in PBSO, stock solution 5 mg/mL for final concentration of 0.002 mg/mL), or with an equivalent amount of PBSO as control for 30 h.

### 2.5. RNA Isolation, Generation of Standard Plasmids and RT-PCR

Total RNA from organoids was extracted by EXTRACTME Total RNA Kit (blirt S.A., Hilden, Germany) and complementary cDNA was synthesized by Reverse Transcription System (Promega, Madison, WI, USA). Standard plasmids were generated using the TOPO TA Cloning^®^Kit For Sequencing (lifetechnologies™, Carlsbad, CA, USA) to analyze absolute gene expression. Amplification of target genes was performed, followed by a transfection into a plasmid vector and transformation into competent One Shot^®^TOP10 + DH5α™-T1^®^ cells (Invitrogen, Carlsbad, CA, USA). Plasmid DNA was characterized by sequencing (GATC Biotech AG, Konstanz, Germany).

RT-PCR was performed as previously described [13]. The oligonucleotide primer sequences are listed in Table S1. To determine absolute gene expression of α-defensin (*Defa*) 1, *Defa21*, *Defa5*, murine β-defensin (*mbD*) 1, lysozyme (*Lyz1*), regenerating islet-derived protein 3 gamma (*Reg3γ*), *Nod2*, and matrix metalloproteinase 7 (*Mmp7*), quantitative standard curves were generated by serial dilution of plasmid standards and normalized to the copy numbers of mouse housekeeping gene β-Actin. Relative gene expression of *ZO-1*, junctional adhesion molecule (*JAM*) A, *Cldn2*, *Cldn5*, *Cldn7*, *Ocln*, mucin (*Muc*)1, *Muc2*, *IL-6*, *IL-1β*, myeloid differentiation primary response 88 (*Myd88*), and tumor necrosis factor α (*Tnfα*) was calculated by comparison to the housekeeping gene β-Actin using the ΔΔ-Ct method.

### 2.6. Statistical Analysis

Statistical analyses were performed using GraphPad Prism software 7.0 (GraphPad Software Inc., La Jolla, CA, USA). Analysis of normal distribution was performed using the Kolmogorov–Smirnov test, with outliers identified using the ROUT method (Q = 1 %). A one-way analysis of variance (ANOVA) with Dunnett’s multiple comparisons test or Kruskal–Wallis test with Dunn’s multiple comparisons test was performed for statistical comparison of more than two groups. Differences between two groups were tested by unpaired t-test or Mann–Whitney test. Data are presented as mean ± standard error of the mean (SEM). *p*-values of <0.05 were considered statistically significant. A statistical trend was defined as 0.05 > *p*-value < 0.1. Correlation analyses were performed by two-tailed Spearman rank correlation, with co-efficients in the range of 0.0 to 0.2 (0.0 to −0.2) defined as no correlation, in the range of 0.2 to 0.4 or −0.2 to −0.4 defined as weak positive or negative correlation, in the range of 0.4 to 0.6 or −0.4 to −0.6 defined as moderate positive or negative correlation, in the range of 0.6 to 0.8 or −0.6 to −0.8 defined as strong positive or negative correlation, in the range of 0.8 to 1.0 or −0.8 to −1.0 defined as very strong positive or negative correlation.

## 3. Results

### 3.1. Dose-Finding Studies

**Figure 1:**
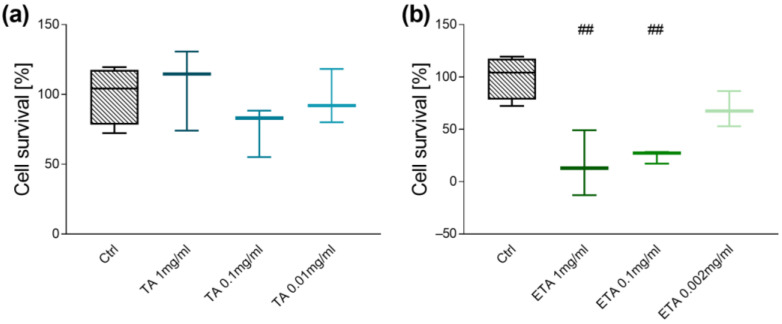
Cell survival of organoids treated with TA (1 mg/mL, 0.1 mg/mL, 0.01 mg/mL) (**a**), or Eta (1 mg/mL, 0.1 mg/mL, 0.002 mg/mL) (**b**), or with PBSO as control for 30 h (n = 3). Data are presented as means ± SEM and were analyzed by one-way ANOVA with Dunnett’s multiple comparisons test or Kruskal–Wallis test with Dunn’s multiple comparisons test. Differences between two groups were analyzed by using unpaired *t*-test or Mann–Whitney test. Significant differences to PBSO control are indicated as ## *p*-value < 0.01.

We first conducted an MTT assay, to exclude cytotoxic effects of TA and Eta stimulation using three different concentrations (TA 1, 0.1, and 0.01 mg/mL, or Eta 1, 0.1, 0.002 mg/mL). TA up to 1 mg/mL and Eta up to 0.1 mg/mL did not affect viability of small intestinal organoids whereas exposure of organoids to Eta at higher concentrations resulted in a reduction of cell survival (*p* < 0.01, Figure 1a,b). Further, ED50 and ED100 calculations based on the MTT assay measurements determined an ED50 of 1.623 mg/mL and an ED100 of 1.787 mg/mL for TA as well as an ED50 of 0.081 mg/mL and an ED100 of 0.087 mg/mL for Eta (Table S2).

**Figure 2:**
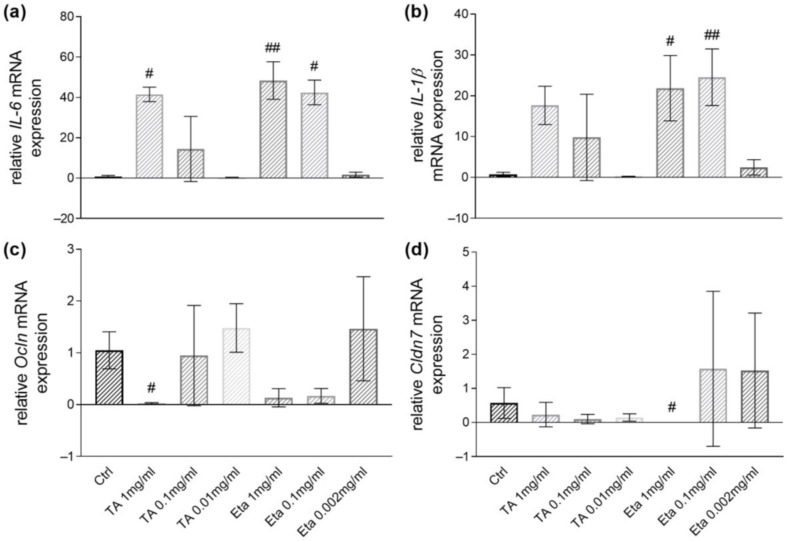
Relative mRNA expression levels of *IL-6* (**a**), *IL-1β* (**b**), *Ocln* (**c**), and *Cldn7* (**d**) determined by quantitative RT-PCR are shown upon stimulation of organoids with TA (1 mg/mL, 0.1 mg/mL, 0.01 mg/mL), or Eta (1 mg/mL, 0.1 mg/mL, 0.002 mg/mL), or with PBSO as control for 30 h. Data are presented as means ± SEM (n = 4). Statistical analysis was performed by one-way ANOVA with Dunnett’s multiple comparisons test or Kruskal–Wallis test with Dunn’s multiple comparisons test. Differences between two groups were analyzed by using unpaired *t*-test or Mann–Whitney test. Significant differences to PBSO control are indicated as # *p*-value < 0.05; ## *p*-value < 0.01.

Consistently, examinations of inflammatory markers by RT-PCR demonstrated that TA up to 0.01 mg/mL and Eta up to 0.002 mg/mL did not induce *IL-1β* or *IL-6* mRNA expression in small intestinal organoids. At higher concentrations, 1 mg/mL TA (*p* < 0.05), 1 mg/mL Eta (*p* < 0.01), and 0.1 mg/mL Eta (*p* < 0.05) induced *IL-6* expression (Figure 2a). Further, organoid stimulation with 1 mg/mL Eta (*p* < 0.05) and 0.1 mg/mL Eta (*p* < 0.01) upregulated *IL-1β* mRNA expression (Figure 2b).

Likewise, PCR analyses showed that stimulation of organoids with TA up to 0.01 mg/mL and Eta up to 0.002 mg/mL did not impair markers of barrier function such as *Ocln* or *Cldn7* mRNA expression, whereas higher concentration of TA or Eta resulted in decreased expressions of TJ genes (Figure 2c,d). In conclusion, these examinations demonstrated that TA at concentrations of 0.01 mg/mL and Eta at concentrations of 0.002 mg/mL are appropriate for use in organoid cell culture. Thus, TA and Eta have been administered in a 5:1 ratio, as in the Tannacomp^®^ formulation for human use that contains TA (250 mg) and Eta (50 mg).

### 3.2. GF_Red_ and LPS Exposure Induce Proinflammatory Cytokine Transcripts Expression in Small Intestinal Organoids

Exposure of organoids to modified CCM formulation (½ R-Spondin, ½ noggin; GF_Red_) for 30 h induced *Myd88* (*p* < 0.01), *Tnfα* (*p* < 0.01), *IL-6* (*p* < 0.05), and *IL-1β* gene expression (*p* < 0.01), indicating inflammatory effects (Table 2). In addition, exposure to LPS also resulted in an increase of several inflammatory markers. Exposure to LPS at 100 µg/mL and 50 µg/mL induced *Myd88* gene expression (*p* < 0.05), whereas *Tnfα* was found to be induced only by exposure with 50 µg/mL LPS (*p* < 0.05). Nevertheless, both 100 µg/mL (*p* < 0.0001) and 50 µg/mL of LPS (*p* < 0.05) increased *IL-6* and *IL-1β* gene expression (*p* < 0.05, Table 2). Comparatively, both GF_Red_ (*p* < 0.05) as well as with 100 µg/mL (*p* < 0.05) or 50 µg/mL LPS (*p* < 0.01) reduced *Ocln* gene expression. Further, organoid treatment with GF_Red_ resulted in a decrease of *Cldn7* expression (*p* < 0.05, Table 2).

### 3.3. TA and Eta Stimulation Improve Stress-Induced Myd88 and Cytokine Transcript Expression

While exposure of organoids to 0.01 mg/mL TA or 0.002 mg/mL Eta had no effects on proinflammatory cytokine transcripts expression (Figure S1), assessment revealed that reduction of growth factors led to an induction of *Myd88* mRNA expression (*p* < 0.001, Table 3). However, this GF_Red_-mediated effect was absent when organoids were co-exposed to 0.01 mg/mL TA (*p* < 0.01) or 0.002 mg/mL Eta (*p* < 0.001), suggesting that both substrates might display anti-inflammatory effects (Table 3). In accordance therewith, reduction of growth factors resulted in an increase of *Tnfα* mRNA expression (*p* < 0.01), whereas co-stimulation with TA (*p* < 0.01) or Eta (*p* < 0.01) at the concentrations indicated above prevented this effect (Table 3). Moreover, GF_Red_-induced stress increased *IL-6* (*p* < 0.01) and *IL-1β* (*p* < 0.01), whereas exposure with LPS had no effects (Table 3). Further, stimulation of GF_Red_-treated organoids with TA or Eta was found to decrease *IL-6* (*p* < 0.05) and *IL-1β* gene expression (*p* < 0.01, Table 3).

### 3.4. TA and Eta Attenuate GF_Red_- and LPS-Induced Gut Barrier Dysfunctions

#### 3.4.1. Stimulation with TA and Eta Improve TJ and Muc Gene Expression in Stressed Organoids

**Figure 3:**
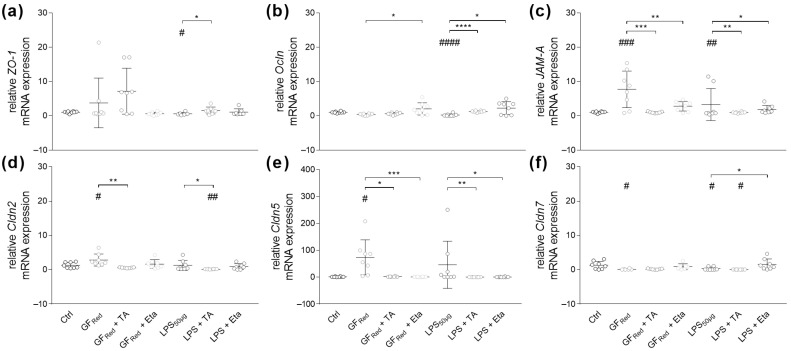
GF_Red_- and LPS-induced stress causes multiple disturbances in TJ and AJ gene expression, whereas TA at 0.01 mg/mL and Eta at 0.002 mg/mL partially reverse these effects. Organoids were treated as previously described in Table 3. Relative mRNA expression levels of *ZO-1* (**a**), *Ocln* (**b**), *JAM-A* (**c**), *Cldn2* (**d**), *Cldn5* (**e**), and *Cldn7* (**f**) were determined by quantitative RT-PCR. Data are presented as means ± SEM (n = 8). Statistical analysis was performed by one-way ANOVA with Dunnett’s multiple comparisons test or Kruskal–Wallis test with Dunn’s multiple comparisons test. Differences between two groups were analyzed by using unpaired *t*-test or Mann–Whitney test. Significant differences to PBSO control are indicated as # *p*-value < 0.05; ## *p*-value < 0.01; ### *p*-value < 0.001; #### *p*-value < 0.0001. Significant differences between two groups are indicated as * *p*-value < 0.05; ** *p*-value < 0.01; *** *p*-value < 0.001; **** *p*-value < 0.0001.

Treating organoids with TA or Eta revealed no effects on *TJ* or *AJ* gene expression (Figure S2). However, LPS stimulation reduced *ZO-1* mRNA expression (*p* < 0.05, Figure 3a). This effect was absent when organoids were exposed to TA (*p* < 0.05, Figure 3a). Consistently, stress induction by GF_Red_ showed no effects on Ocln gene expression, whereas LPS decreased *Ocln* mRNA expression (*p* < 0.0001, Figure 3b). Additionally, these effects were no longer present when organoids were exposed to TA (*p* < 0.0001) or Eta (*p* < 0.05, Figure 3b). Stress induction by GF_Red_ (*p* < 0.001) as well as by LPS (*p* < 0.01) resulted in an induction of JAM-A gene expression (Figure 3c), whereby concomitant stimulation with TA (*p* < 0.001) or Eta (*p* < 0.01) prevented GF_Red_-mediated effects (Figure 3c). Similarly, both TA (*p* < 0.01) and Eta (*p* < 0.05) were found to attenuate LPS-dependent changes in JAM-A gene expression (Figure 3c).

Our data revealed that stress induction by GF_Red_ increased *Cldn2*, *Cldn5* and decreased *Cldn7* (*p* < 0.05) gene expression, whereas LPS exposure affected *Cldn7* mRNA expression (*p* < 0.05, Figure 3d–f). Moreover, *Cldn2* (*p* < 0.01) and *Cldn5* (*p* < 0.05) gene expression was normalized when GF_Red_-treated organoids were additionally exposed to TA. Similarly, TA reduced gene expression of *Cldn2* (*p* < 0.01) and *Cldn5* (*p* < 0.01) in LPS-treated cells (Figure 3d,e). Moreover, stimulation with Eta improved *Cldn5* expression (*p* < 0.001) in GF_Red_-treated organoids, as well as *Cldn5* and *Cldn7* expression (*p* < 0.05) in LPS-stimulated cells (Figure 3d–f).

**Figure 4:**
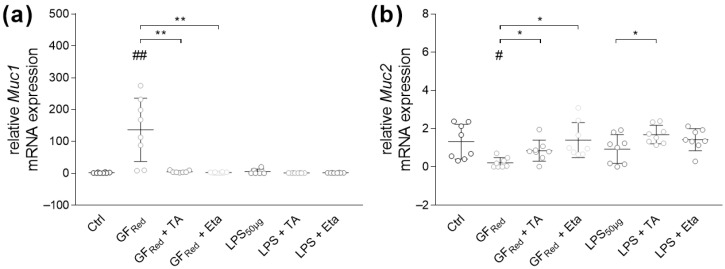
GF_Red_ impairs mucus transcripts expression, with both TA at 0.01 mg/mL and Eta at 0.002 mg/mL normalizing these effects. Organoids were treated as previously described in Table 3. Relative mRNA expression levels of *Muc1* (**a**) and *Muc2* (**b**) were determined by quantitative RT-PCR. Data are presented as means ± SEM (n = 8). Statistical analysis was performed by one-way ANOVA with Dunnett’s multiple comparisons test or Kruskal–Wallis test with Dunn’s multiple comparisons test. Differences between two groups were analyzed by using unpaired *t*-test or Mann–Whitney test. Significant differences to PBSO control are indicated as # *p*-value < 0.05; ## *p*-value < 0.01. Significant differences between two groups are indicated as * *p*-value < 0.05; ** *p*-value < 0.01.

Further examinations revealed that GF_Red_-induced stress was associated with an increase in *Muc1* (*p* < 0.01) and a decrease in *Muc2* mRNA expression (*p* < 0.05), whereas LPS had no effects (Figure 4a,b). Although organoids treated with TA exhibited increased *Muc1* gene expression (*p* < 0.05, Figure S3), stimulation of GF_Red_-treated organoids with TA or Eta resulted in a reduction of stress-induced effects, which was characterized by a decrease in *Muc1* (*p* < 0.01) and an increase in *Muc2* (*p* < 0.05) gene expression (Figure 4a,b).

#### 3.4.2. TA and Eta Regulate Antimicrobial Peptide Gene Expression in GF_Red_- and LPS-Treated Organoids

**Figure 5:**
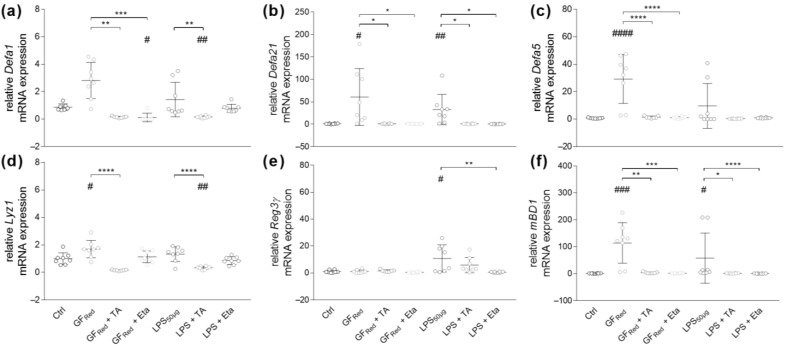
GF_Red_- and LPS-induced stress is associated with increased antimicrobial peptide transcripts expression, which is normalized by co-stimulation with TA at 0.01 mg/mL or Eta at 0.002 mg/mL. Organoids were treated as previously described in Table 3. Relative mRNA expression levels of Defa1 (**a**), Defa21 (**b**), Defa5 (**c**), Lyz1 (**d**), Reg3γ (**e**), and mBD1 (**f**) were determined by quantitative RT-PCR. Data are presented as means ± SEM (n = 8). Statistical analysis was performed by one-way ANOVA with Dunnett’s multiple comparisons test or Kruskal–Wallis test with Dunn’s multiple comparisons test. Differences between two groups were analyzed by using unpaired *t*-test or Mann–Whitney test. Significant differences to PBSO control are indicated as # *p*-value < 0.05; ## *p*-value < 0.01; ### *p*-value < 0.001; #### *p*-value < 0.0001. Significant differences between two groups are indicated as * *p*-value < 0.05; ** *p*-value < 0.01; *** *p*-value < 0.001; **** *p*-value < 0.0001.

Since AMPs have been identified as an important intestinal defense strategy, we next evaluated AMP gene expression. Examinations revealed no effects of TA or Eta exposure in organoids on *Defa1*, *Defa21*, *Defa5*, *Lyz1* or *Reg3γ* transcripts expression (Figure S4). Although GF_Red_-mediated stress did not affect *Defa1* gene expression, GF_Red_-treated organoids exhibited increased *Defa21* (*p* < 0.01) and *Defa5* (*p* < 0.0001) gene expression (Figure 5a–c). Both *Defa21* (*p* < 0.05) and *Defa5* (*p* < 0.0001) expression were normalized when organoids were concomitantly exposed to TA or Eta (Figure 5b,c). Furthermore, LPS exposure resulted in an induction of *Defa21* expression (*p* < 0.05, Figure 5b), whereas these effects were absent when organoids were co-exposed to TA (*p* < 0.05) or Eta (*p* < 0.05, Figure 5b). Analysis of other AMPs revealed that treatment of organoids with GF_Red_ induced the expression of *Lyz1* (*p* < 0.05) and *mBD1* (*p* < 0.001). Similarly, LPS exposure was associated with an increase in *Reg3γ* and *mBD1* gene expression (*p* < 0.05, Figure 5d–f). Although TA exposure increased *mBD1* gene expression in organoids, co-stimulation of GF_Red_-treated organoids with TA reduced *Lyz1* (*p* < 0.0001) and *mBD1* gene expression (*p* < 0.01, Figure 5d,f). Further, treatment of LPS-stimulated organoids with both TA (*p* < 0.05) and Eta (*p* < 0.0001) decreased mRNA expression of *mBD1*. Furthermore, *Reg3γ* expression was normalized when organoids were co-exposed to Eta (*p* < 0.01, Figure 5e,f).

**Figure 6:**
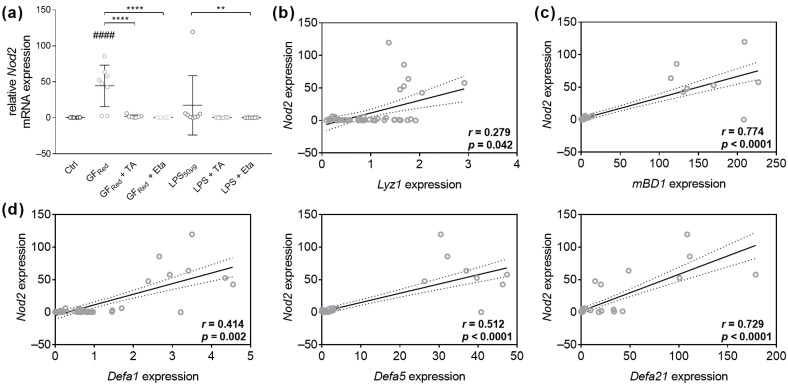
TA and Eta reduce Nod2-dependent activation of antimicrobial peptide gene expression in GF_Red_-treated cells. Organoids were treated with TA at 0.01 mg/mL and Eta at 0.002 mg/mL as previously described in Table 3. Relative mRNA expression levels of Nod2 (**a**) were determined by quantitative RT-PCR. Data are presented as means ± SEM (n = 8). Statistical analysis was performed by one-way ANOVA with Dunnett’s multiple comparisons test or Kruskal–Wallis test with Dunn’s multiple comparisons test. Differences between two groups were analyzed by using unpaired *t*-test or Mann–Whitney test. Significant differences to PBSO control are indicated as #### *p*-value < 0.0001. Significant differences between two groups are indicated as ** *p*-value < 0.01; **** *p*-value < 0.0001. Correlation analysis for Nod2 gene expression and Lyz1 (**b**), mBD1 (**c**), Defa1, Defa5, and Defa21 (**d**) expression was performed by two-tailed Spearman rank correlation analysis. Correlations were defined as follows: 0.2 to 0.4, weak positive correlations; 0.4 to 0.6, moderate positive correlations; 0.6 to 0.8, strong positive correlations.

GF_Red_-mediated stress in organoids increased *Nod2* mRNA expression (*p* < 0.0001), whereas LPS exposure had no effects (Figure 6a). Interestingly, co-stimulation of GF_Red_-treated organoids with TA or Eta was found to reduce *Nod2* gene expression (*p* < 0.0001, Figure 6a), while exposure of organoids to TA or Eta revealed no effects on Nod2 gene expression (Figure S5). While Spearman rank correlation analysis revealed no correlation between *Nod2* and *Reg3γ* (Figure S6), there was a weak positive correlation between *Nod2* mRNA levels and *Lyz1* gene expression (r = 0.279, *p* = 0.042, Figure 6b). Further, there was a moderate positive correlation between *Nod2* and the expression of *Defa1* (r = 0.414; *p* = 0.002), and *Defa5* (r = 0.512; *p* < 0.0001, Figure 6d) and a strong positive correlation between the expression of *Nod2* and *mBD1* (r = 0.774; *p* < 0.0001) as well as *Defa21* expression (r = 0.729; *p* < 0.0001, Figure 6c,d).

**Figure 7:**
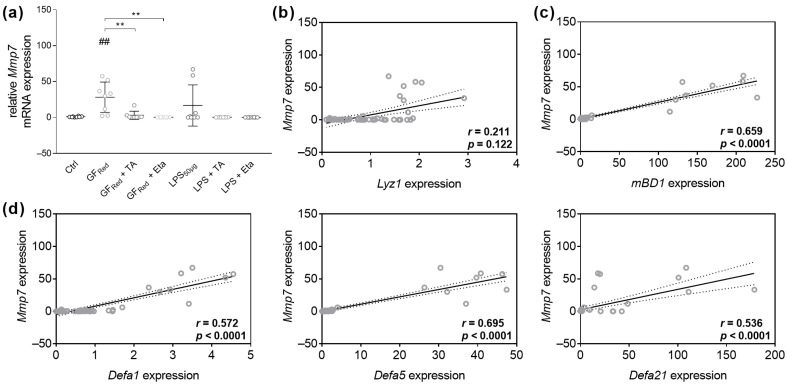
Increased Mmp7 expression in GF_Red_-treated cells correlates with elevated AMP formation. Organoids were treated as previously described in Table 3. Relative mRNA expression levels of Mmp7 (**a**) were determined by quantitative RT-PCR. Data are presented as means ± SEM (n = 8). Statistical analysis was performed by one-way ANOVA with Dunnett’s multiple comparisons test or Kruskal–Wallis test with Dunn’s multiple comparisons test. Differences between two groups were analyzed by using unpaired *t*-test or Mann–Whitney test. Significant differences to PBSO control are indicated as ## *p*-value < 0.01. Significant differences between two groups are indicated as ** *p*-value < 0.01. Correlation analysis for Mmp7 gene expression and Lyz1 (**b**), mBD1 (**c**), Defa1, Defa5, and Defa21 (**d**) expression was performed by two-tailed Spearman rank correlation analysis. Correlations were defined as follows: 0.2 to 0.4, weak positive correlations; 0.4 to 0.6, moderate positive correlations; 0.6 to 0.8, strong positive correlations.

Additionally, measurements demonstrated that stress conditions caused by GF_Red_ resulted in an increase of *Mmp7* expression (*p* < 0.01), whereas this effect was absent when organoids were co-incubated with TA or Eta (*p* < 0.01). Further, organoids treated with Eta showed a reduction of Mmp7 transcript expression (*p* < 0.05, Figure S5). In contrast, no effects on *Mmp7* expression were observed under LPS exposure (Figure 7a). We next performed Spearman rank correlation analysis. Examinations demonstrated a weak positive correlation between *Mmp7* and *Lyz1* gene expression (r = 0.211, *p* = 0.122, Figure 7b), as well as moderate positive correlation between *Mmp7* and *Defa1* (r = 0.572; *p* < 0.0001), and *Defa21* (r = 0.536; *p* < 0.0001, Figure 7d). Moreover, we found a strong positive correlation between *Mmp7* and *mBD1* (r = 0.659; *p* < 0.0001) and *Defa5* gene expression (r = 0.659; *p* < 0.0001, Figure 7c,d). However, no correlation was found between *Mmp7* and *Reg3γ* (Figure S6).

**Figure 8:**
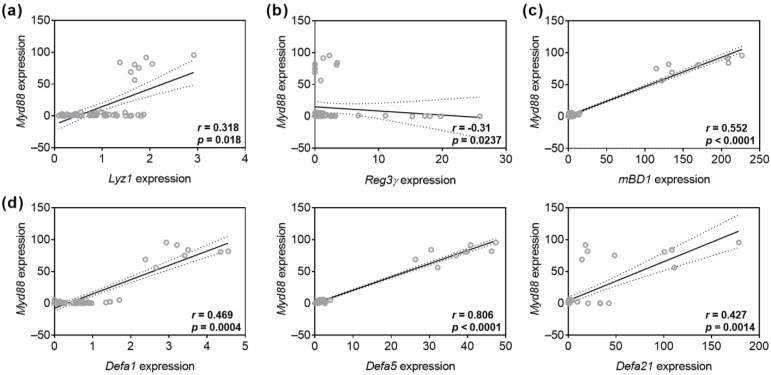
GF_Red_-mediated activation of Myd88 transcripts expression is associated with increased AMP gene expression. Organoids were treated with TA at 0.01 mg/mL and Eta at 0.002 mg/mL as previously described in Table 3. Correlation analysis for Myd88 gene expression and Lyz1 (**a**), Reg3γ (**b**), mBD1 (**c**), Defa1, Defa5, and Defa21 (**d**) expression. Statistical analysis was performed by two-tailed Spearman rank correlation analysis. Correlations were defined as follows: −0.2 to −0.4, weak negative correlations; 0.2 to 0.4, weak positive correlations; 0.4 to 0.6, moderate positive correlations; 0.8 to 1.0, very strong positive correlations.

As antimicrobial peptide defense has been linked with the TLR/Myd88 signaling pathway, we next analyzed potential associations between *Myd88* and AMP mRNA expression. Examinations demonstrated a weak positive correlation between *Myd88* and *Lyz1* gene expression in small intestinal organoids (r = 0.318, *p* = 0.018, Figure 8a). Interestingly, there was a weak negative correlation between the expression of *Myd88* and *Reg3γ* mRNA levels (r = −0.31, *p* = 0.0237, Figure 8b). We also identified a moderate positive correlation between *Myd88* and *mBD1* (r = 0.552; *p* < 0.0001, Figure 8c), *Defa1* (r = 0.469; *p* = 0.0004), and *Defa21* gene expression (r = 0.427, *p* = 0.0014, Figure 8d). Moreover, analysis revealed a very strong positive correlation between *Myd88* and *Defa5* expression (r = 0.806; *p* < 0.0001, Figure 8d).

## 4. Discussion

The present study provides evidence that both TA and Eta improve stress-induced GI barrier dysfunctions, especially by reducing inflammatory cytokine transcripts expression in organoids. These effects were accompanied by an improved TJ, AJ and Muc expression and by a normalized *Nod2*-, *Myd88*-, and *Mmp7*-dependent activation of antimicrobial peptide gene expression. Further, our data also show that exposure to LPS, as well as reducing CCM growth factors R-Spondin and noggin, both regulating the Wnt signaling pathway, is appropriate to induce stress reactions in murine small intestinal organoids.

We were able to show that TA and Eta at selected doses reduced GF_Red_-mediated induction of inflammatory cytokine transcripts expression, indicating anti-inflammatory activity of both substrates. Tannacomp^®^ is a pharmaceutical drug that is commonly used for treating and preventing diarrhea. While its primary effects are derived from antimicrobial and astringent properties [7,36,37], there is evidence that Tannacomp^®^, especially its component TA, might also have anti-inflammatory effects [6]. Our data revealed that stimulation of murine small intestinal organoids with TA inhibited stress-mediated induction of *Myd88*, *Tnfα*, *IL-6* and *IL-1β* gene expression. This is in accordance with the results of further studies. Consistently, stimulation of LPS-treated Caco-2 cells with gelatin tannate, a combination of TA and gelatin, resulted in a dose-dependent inhibition of IL-8 and TNF-α release, as well as a decrease of intercellular adhesion molecule-1 gene expression [38]. In addition, TA treatment of LPS-stimulated BV2 microglial cells resulted in a reduction of IL-6, IL-1β and TNF-α protein levels by suppressing the nuclear factor kappa-light-chain-enhancer of activated B-cells (NF-κB) signaling pathway, indicating anti-neuroinflammatory effects [39]. Similarly, TA was found to specifically inhibit CXC motif chemokine 12 (CXCL12)-induced migration of human monocytes as well as CXCL12 binding to THP-1 cells and CXCL12-induced migration of MDA-231 breast tumor cells in vitro, providing a possible underlying mechanism for its reported anti-inflammatory activity [40]. Accordingly, oral TA treatment of mice with ethanol-induced and ethanol/HCl-induced gastric ulcers exhibited gastroprotective effects, which were associated with a decrease in TNF-α, IL-1β and IL-6 protein levels and an increase in IL-10 protein levels in gastric tissue. Further potassium channels and production of sulfhydryl compounds, nitric oxide (NO), and prostaglandin E2 (PGE2) were involved in these effects [41]. TA has been found to exhibit a potential role in IL-1β-related diseases, such as osteoarthritis (OA), by preventing IL-1β–IL-1R1 interactions and inhibiting IL-1β-induced expression of *IL-6*, *Tnfα*, and *PGE2*, as well as IL-1β-induced mitogen-activated protein kinases (MAPK) and NF-κB activation in human OA chondrocytes. In addition, TA reduced pain, cartilage degradation, and IL-1β-mediated inflammation in a monosodium iodoacetamide-induced OA model in rats [42].

We demonstrated for the first time that Eta downregulates mRNA expression of proinflammatory markers such as *Myd88*, *Tnfα*, *IL-6* and *IL-1β* in small intestinal organoids. There is evidence that Eta indirectly reduced inflammatory reactions caused by bacterial toxins through its antibacterial and antiseptic effects. While studies of antibiotic susceptibility and bactericidal time revealed only moderate activity of Eta against *Pseudomonas aeruginosa* [43], antibacterial efficacy of two-dimensional graphene-based nanocarriers loaded with sulfamethoxazole and Eta against several Gram-negative and -positive bacterial isolates has been reported [7]. Thus, Eta nanoformulation exhibited bactericidal activities against *Escherichia coli K1*, *Serratia marcescens*, *Pseudomonas aeruginosa* and *Salmonella enterica* and showed less toxic effects against human cells [7]. Furthermore, a recent study indicated that Eta provided antiviral activity and could be a potential inhibitor of SARS-CoV-2, as Eta inactivated virus particles in human primary nasal epithelial cells [44].

Our results revealed that TA and Eta attenuated TJ and AJ expression and normalized mucus transcripts expression in murine small intestinal organoids exposed to stress conditions. In accordance with our results, oral TA administration in an oxidative stress mouse model improved jejunal villus height and crypt depth. In addition, TA modulated the GI barrier by inhibiting jejunal claudin and inducing *ZO-1* mRNA expression [8]. Comparable effects were observed in a rat diarrhea model with 10 days of oral TA treatment, resulting in a preservation of intestinal mucosal structure and ZO-1 protein formation, as well as in a selective decrease of interferon-γ (INF-γ) and transforming growth factor-beta [33]. TA further improves mucosal resistance. Thus, rats infected with *Salmonella enteritidis* showed reduced infection-related diarrhea and improved intestinal permeability, measured by permeability marker chromium-EDTA, when given additional TA through diet [45]. Furthermore, feeding weaned piglets with TA induced jejunal protein expression of ZO-1, while in vitro experiments additionally revealed that TA attenuated oxidative stress in IPEC-J2 cells by upregulating *ZO-1*, *Ocln*, and *Cldn1* mRNA and protein expression, thereby enhancing transepithelial electrical resistance [46].

While modulatory effects of TA on GI barrier function have already been described, our data further indicate that Eta also exhibited regulatory effects on the GI barrier through modulating TJ, AJ and Muc gene expression. This might be a result of its anti-inflammatory effects. There is evidence that increased inflammatory responses affected intestinal integrity. Thus, a mouse study revealed that claudin-5 expression was JAM-A-dependent regulated via CCAAT/enhancer-binding protein-α, thereby disturbing endothelial permeability [47]. Further, immune signals, including IL-13 and IL-22, led to increased transcription and expression of pore-forming *Cldn2*, thereby increasing pore pathway permeability and water influx into the intestinal lumen [48]. Similarly, *Citrobacter rodentium*-infected mice exhibited increased permeability, which was associated with IL-22-dependent upregulation of claudin-2 protein expression in the colon [49]. It has been demonstrated that *Muc1* gene expression was also induced by proinflammatory cytokines such as TNF-α, IL-6, IL-1β and IL-22 during inflammatory processes [50], indicating a key role for host pathogen defenses during infections [51].

Further, we demonstrated that organoid cell stress caused by GF_Red_ led to *Nod2*-, *Myd88*-, and *Mmp7*-mediated induction of AMP gene expression, whereas both TA and Eta stimulation were found to normalize antimicrobial peptide expression. TA and Eta have been described as having antimicrobial activity against bacteria and viruses. In vitro, TA inhibited sporulation, toxin production and biofilm formation of *Clostridium difficile* (*C. difficile*) and improved survival and IL-10 serum levels in *C. difficile*-infected C57BL/6N mice [52]. TA was also found to inhibit the growth of intestinal bacteria, including *Clostridium* spp., *Salmonella*, *Bacteroides*, *Enterobacter*, *Lactobacillus* [53], and to be antibacterial against *Staphylococcus aureus* in vitro [54,55,56]. Similarly, an Eta nanoformulation exhibited antibacterial activity against *Escherichia coli K1*, *Serratia marcescens*, *Pseudomonas aeruginosa* and *Salmonella enterica*, as well as against Gram-positive bacteria including *Bacillus cereus*, *Streptococcus pyogenes* and *Streptococcus pneumoniae* in vitro [7]. There is evidence that TA could be a potential candidate for treating norovirus infections and for SARS-CoV-2 viruses, as TA inhibited the entry of SARS-CoV-2 pseudovirus into human hACE2 and Vero E6 cells [57,58]. Several mechanisms have been discussed as potentially involved in TA- and Eta-dependent antimicrobial effects. In addition, there is evidence that TA exhibited the ability to bind through bacterial cell wall peptidoglycan, thus resulting in impaired integrity, but also possibly inhibited efflux pumps, thereby blocking efflux of tetracycline in *Staphylococcus aureus IS-58* and erythromycin in *Staphylococcus aureus RN4220* [56,59].

## 5. Conclusions

In conclusion, our data demonstrated that both TA (0.01 mg/mL) and Eta (0.002 mg/mL) were appropriate for use in murine small intestinal organoids. We were also able to implement a novel method to induce organoid stress responses by reducing CCM growth factors R-Spondin and noggin (GF_Red_). Both TA and Eta stimulation exerted anti-inflammatory effects by reducing gene expression of several inflammatory markers. Moreover, stimulation with TA and Eta improved stress-induced disturbances of TJ, AJ and Muc gene expression and normalized *Nod2*-, *Myd88*-, and *Mmp7*-mediated induction of antimicrobial peptide transcripts expression. Overall, our results provide new evidence that both TA and Eta modulate inflammatory processes and GI barrier function, suggesting that these components might be useful for therapeutic strategies to improve gut barrier function and host defense against GI-infectious diseases.

## Figures and Tables

**Table 1:** Modifications of cell culture medium.

	CCM	GF_Red_
GlutaMax^TM^	2 mM	2 mM
Hepes	10 mM	10 mM
R-Spondin	1 µg/mL	0.5 µg/mL
Noggin	100 ng/µL	50 ng/µL
B-27™ supplement	20 µL/mL	20 µL/mL
N-Acetylcysteine	1.63 mg/mL	1.63 mg/mL
Primocin	0.1 mg/mL	0.1 mg/mL
mEGF	50 ng/mL	50 ng/mL

**Table 2:** Inflammatory and gut barrier transcripts measured in GF_Red_- and LPS-treated organoids.

	Ctrl	GF_Red_	LPS_100µg_	LPS_50µg_
*Myd88*	0.49 ± 0.18	10.25 ± 2.54 ##	10.13 ± 3.76 #	13.15 ± 7.73 #
*Tnfα*	0.53 ± 0.11	25.27 ± 6.52 ##	13.39 ± 9.39	22.45 ± 11.3 #
*IL-6*	0.85 ± 0.29	42.8 ± 11.77 #	8.34 ± 0.46 ####	26.79 ± 14.15 #
*IL-1β*	0.49 ± 0.18	10.25 ± 2.54 ##	10.13 ± 3.76 #	13.15 ± 7.73 #
*Ocln*	1.14 ± 0.17	0.57 ± 0.14 #	0.28 ± 0.1 #	0.17 ± 0.09 ##
*Cldn7*	1.11 ± 0.33	0.16 ± 0.08 #	0.18 ± 0.09	0.68 ± 0.34

Both GF_Red_ and LPS exposure induce stress reactions in small intestinal organoids. Organoids were treated with GF_Red_, or LPS (50 µg, 100 µg), or PBSO as control for 30 h. Gene expression of *Myd88*, *Tnfα*, *IL6*, *IL1β*, *Ocln* and *Cldn7* in small intestinal organoids was determined by quantitative RT-PCR. Data are shown as means ± SEM (n = 4–8). Statistical analysis was performed by one-way ANOVA with Dunnett’s multiple comparisons test or Kruskal–Wallis test with Dunn’s multiple comparisons test. Differences between two groups were analyzed by using unpaired *t*-test or Mann–Whitney test: # *p*-value < 0.05; ## *p*-value < 0.01; #### *p*-value < 0.0001.

**Table 3:** Gene expression of inflammatory markers measured in small intestinal organoids.

	*Myd88*	*Tnfα*	*IL-6*	*IL-1β*
Ctrl	1.11 ± 0.19	1.07 ± 0.14	0.99 ± 0.28	1.64 ± 0.72
GF_Red_	58.05 ± 12.69 ###	46.58 ± 12.02 ##	134.4 ± 36.97 ##	66.39 ± 16.45 ##
GF_Red_ ± TA	1.42 ± 0.37 **	1.8 ± 0.51 **	3.5 ± 0.94 *	3.93 ± 1.41 **
GF_Red_ ± Eta	3.17 ± 0.94 ***	2.21 ± 0.75 **	1.67 ± 0.48 *	4.7 ± 1.39 **
LPS	23.05 ± 14.18	20.87 ± 12.37	42.24 ± 25.97	42.8 ± 28.17
LPS ± TA	0.89 ± 0.15	1.03 ± 0.26	0.33 ± 0.08 $	0.93 ± 0.15
LPS ± Eta	1.82 ± 0.49	1.35 ± 0.4	2.24 ± 0.51	4.73 ± 1.67

GF_Red_-treatment of organoids induces *Myd88* as well as proinflammatory cytokines transcripts expression, with both TA and Eta reversing these effects. Organoids were treated with TA (0.01 mg/mL), or Eta (0.002 mg/mL), or GF_Red_ ± 0.01 mg/mL TA, or GF_Red_ ± 0.002 mg/mL Eta, or 50 µg/mL LPS ± 0.01 mg/mL TA, or 50 µg/mL LPS ± 0.002 mg/mL ETA, or PBSO as control for 30 h. mRNA expression of *Myd88*, *Tnfα*, *IL-6*, and *IL-1β* in small intestinal organoids was determined by quantitative RT-PCR. Data are shown as means ± SEM (n = 8). Statistical analysis was performed by one-way ANOVA with Dunnett’s multiple comparisons test or Kruskal–Wallis test with Dunn’s multiple comparisons test. Differences between two groups were analyzed by using unpaired *t*-test or Mann–Whitney test: Significant differences to PBSO control are indicated as ## *p*-value < 0.01; ### *p*-value < 0.001. Significant differences to GF_Red_ are indicated as * *p*-value < 0.05; ** *p*-value < 0.01; *** *p*-value < 0.001. Significant differences to LPS are indicated as $ *p*-value < 0.05.

## Data Availability

The data presented in this study are available upon justified request to the corresponding author.

## Supplementary Materials

The following supporting information can be downloaded at: https://www.mdpi.com/article/10.3390/biom15050650/s1, Figure S1: Exposure of organoids to TA or Eta has no effects on proinflammatory cytokine transcripts expression; Figure S2: Exposure of organoids to TA increased Cldn5 gene expression; Figure S3. Exposure of organoids to TA increased Muc1 gene expression; Figure S4: TA exposure is associated with increased mBD1 gene expression; Figure S5: Eta exposure is associated with decreased Mmp7 gene expression; Figure S6: Correlation analysis for *Nod2* (a) or *Mmp7* (b) and *Reg3γ* mRNA expression; Table S1: Primers used for RT-PCR; Table S2: Optical density measured by MTT assay for calculating ED50 and ED100 for TA and Eta.

## Abbreviations

The following abbreviations are used in this manuscript:

Eta	Ethacridine lactate
TA	Tannic acid
LPS	Lipopolysaccharide
GF_Red_	CCM with reduced growth factors
GI	Gastrointestinal
TJ	Tight junction
AJ	Adherent junction
IBD	Inflammatory bowel disease
CD	Crohn’s disease
Ocln	Occludin
ZO-1	Zonula occludens 1
Cldn	Claudin
AMP	Antimicrobial Peptide
TLR	Toll-like receptor
SCFA	Short-chain fatty acid
CIB	Crypt isolation buffer
CCM	Cell culture medium
IL	Interleukin
Defa	α-defensin
mBD1	Murine β-defensin 1
Lyz1	Lysozyme
Reg3γ	Regenerating islet-derived protein 3 gamma
Mmp7	Matrix metalloproteinase-7
JAM-A	Junctional adhesion molecule A
Muc	Mucin
Myd88	Myeloid differentiation primary response 88
Tnfα	Tumor necrosis factor α
ANOVA	One-way analysis of variance
SEM	Standard error of the mean
Ctrl	PBSO control
NF-κB	Nuclear factor kappa-light-chain-enhancer of activated B-cells ()
CXCL12	CXC motif chemokine 12
NO	Nitric oxide
PGE2	Prostaglandin 2
OA	Osteoarthritis
MAPK	Mitogen-activated protein kinases
INF-γ	Interferon-γ
HCN	Hepatitis C virus
