# Supplementary material for: Tannic Acid and Ethacridine Lactate Attenuate Markers of Stress-Induced Intestinal Barrier Dysfunctions in Murine Small Intestinal Organoids
Source: Biomolecules. 2025 Apr 30;15(5):650. doi: 10.3390/biom15050650 (PMC12109227; doi:10.3390/biom15050650)
Supplement: Supplementary file 1 [file biomolecules-15-00650-s001.zip › biomolecules-3540710-supplementary.pdf]

Supplements

Table S1. Primers used for RT-PCR.

Primer	Forward (5′-3′)	Reverse (5′-3′)
Actb	GCTGAGAGGGAAATCGTGCGTG	CCAGGGAGGAAGAGGATGCGG
Defa1	TCAAGAGGCTGCAAAGGAAGAGAAC	TGGTCTCCATGTTCAGCGACAGC
Defa5	TCAAAAAAGCTGATATGCTATTG	AGCTGCAGCAGAATACGAAAG
Defa21	CCAGGGGAAGATGACCAGGCT	TGCAGCGACGATTTCTACAAAGGC
Lyz1	GCCAAGGTCTACAATCGTTGTGAGTTG	CAGTCAGCCAGCTTGACACCACG
Reg3g	TTCCTGTCCTCCATGATCAAAA	CATCCACCTCTGTTGGGTTCA
mBD1	CCAGGCTGCCCATCTAATACC	ATTTCCTGCTCGCTACCTAA
Mmp7	TTCAAGAGGGTTAGTTGGGGGACTG	CCGCCTCTACGAGTGAAACTGTT
Nod2	GGCACCTGAAGTTGACATTTTGC	ATCTCCCACAGAGTTGTAATCC
Muc1	TCCTTCTGAGAGCCACCACT	GGTGACCACTTCTGCCAACT
Muc2	GATGGCACCTACCTCGTTGT	GTCCTGGCACTTGTTGGAAT
Cldn2	GTCATCGCCCATCAGAAGAT	ACTGTTGGACAGGGAACCAG
Cldn5	GCTCTCAGAGTCCGTTGACC	CTGCCCTTTCAGGTTAGCAG
Cldn7	GCGACAACATCATCACAGCC	CCTTGGAGGAATTGGACTTGG
JAM-A	CACCTTCTCATCCAGTGGCATC	CTCCACAGCATCCATGTGTGC
Ocln	ACTCCTCCAATGGACAAGTG	CCCCACCTGTCGTGTAGTCT
ZO-1	CCACCTCTGTCCAGCTCTTC	CACCGGAGTGATGGTTTTCT
IL-6	AGTCACAGAAGGAGTGGCTA	CTGACCACAGTGAGGAATGT
IL-1β	ACGGATTCCATGGTGAAGTC	GAGTGTGGATCCCAAGCAAT
Myd88	CAAAAGTGGGGTGCCTTTGC	AAATCCACAGTGCCCCCAGA
Tnfα	ACCACCATCAAGGACTCA	AGGTCTGAAGGTAGGAAG

Abbreviation: *Actb*, β-Actin; *Defa*, α-defensin; *Lyz1*, Lysozyme; *Reg3g*, Regenerating islet-derived protein 3 gamma; *mBD1*, Murine β-defensin 1; *Mmp7*, Matrix metalloproteinase-7; *Nod2*, Nucleotide binding oligomerization domain; *Muc*, Mucin; *Cldn*, Claudin; *JAM-A*, Junctional adhesion molecule A; *Ocln*, Occludin, *ZO-1*, zonula occludens 1; *IL*, Interleukin; *Myd88*, Myeloid differentiation primary response 88; *Tnfα*, Tumor necrosis factor α.

Table S2. Optical densitiy measured by MTT assay for calculating ED50 and ED100 for TA and Eta.

	Sample 1	Sample 2	Sample 3
**PBSO Ctrl**	0.446	0.289	0.478
**TA 10 mg/ml**	1.551	1.538	1.957
**TA 1 mg/ml**	0.296	0.458	0.523
**TA 0.1 mg/ml**	0.332	0.353	0.22
**TA 0.01 mg/ml**	0.32	0.473	0.368
**Eta 10 mg/ml**	0.279	0.394	0.744
**Eta 1 mg/ml**	-0.051	0.196	0.052
**Eta 0.1 mg/ml**	0.069	0.109	0.113
**Eta 0.01 mg/ml**	0.355	0.498	0.532
**Eta 0.002 mg/ml**	0.27	0.212	0.346

Figure S1

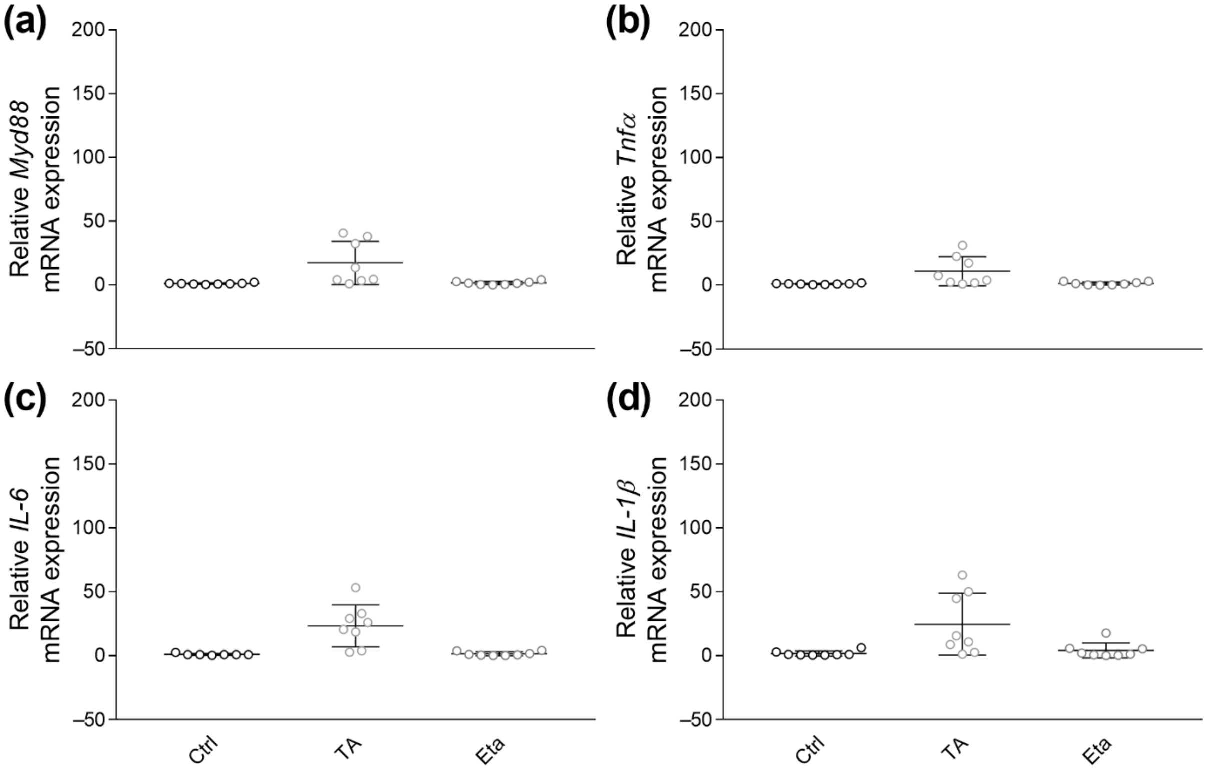

Figure S1. Exposure of organoids to TA or Eta has no effects on proinflammatory cytokine transcripts expression. Organoids were treated with TA (0.01 mg/ml), or Eta (0.002 mg/ml), or PBSO as control for 30 h. mRNA expression of *Myd88* **(a)**, *Tnfα* **(b)**, *IL-6* **(c)**, and *IL-1β* **(d)** in small intestinal organoids determined by quantitative RT-PCR. Data are shown as means ± SEM (n = 8). Statistical analysis was performed by one-way ANOVA with Dunnett's multiple comparisons test or Kruskal-Wallis test with Dunn's multiple comparisons test. Differences between two groups were analyzed by using unpaired t-test or Mann–Whitney test.

Figure S2

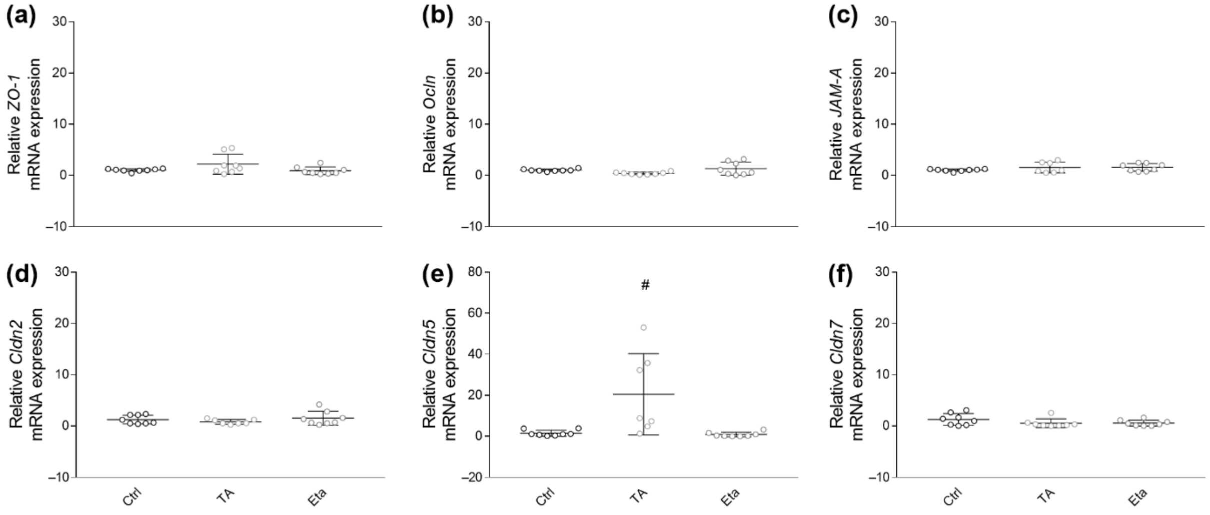

Figure S2. Exposure of organoids to TA increased *Cldn5* gene expression. Organoids were treated as previously described in Figure S1. Relative mRNA expression levels of *ZO-1* **(a)**, *Ocln* **(b)**, *JAM-A* **(c)**, *Cldn2* **(d)**, *Cldn5* **(e)**, and *Cldn7* **(f)** were determined by quantitative RT-PCR. Data are presented as means ± SEM (n = 8). Statistical analysis was performed by one-way ANOVA with Dunnett's multiple comparisons test or Kruskal-Wallis test with Dunn's multiple comparisons test. Differences between two groups were analyzed by using unpaired t-test or Mann–Whitney test. Significant differences to PBSO control are indicated as #p-value < 0.05.

Figure S3

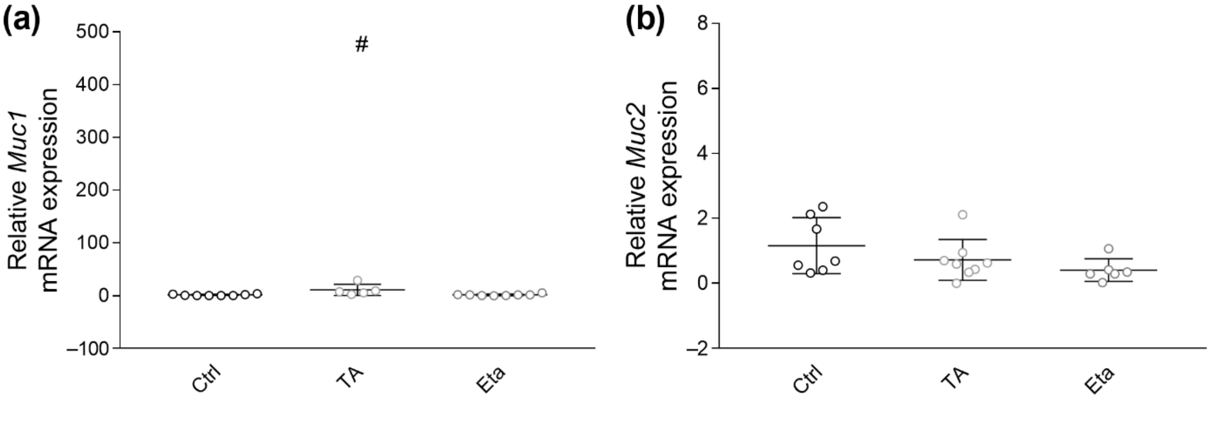

Figure S3. Exposure of organoids to TA increased *Muc1* gene expression. Organoids were treated as previously described in Figure S1. Relative mRNA expression levels of *Muc1* **(a)** and *Muc2* **(b)** were determined by quantitative RT-PCR. Data are presented as means ± SEM (n = 8). Statistical analysis was performed by one-way ANOVA with Dunnett's multiple comparisons test or Kruskal-Wallis test with Dunn's multiple comparisons test. Differences between two groups were analyzed by using unpaired

t-test or Mann–Whitney test. Significant differences to PBSO control are indicated as #p-value < 0.05.

Figure S4

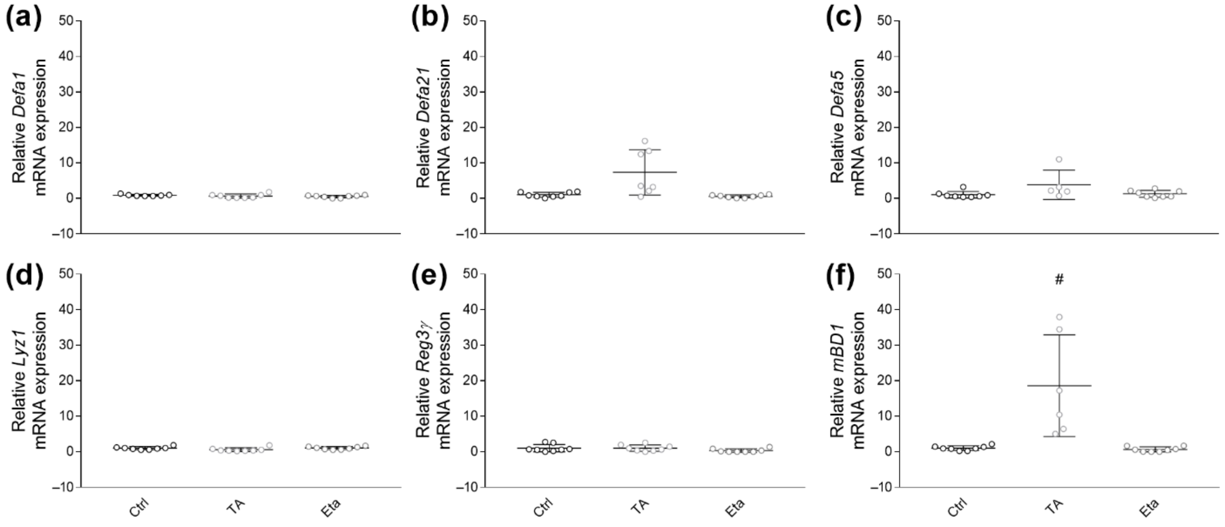

Figure S4. TA exposure is associated with increased *mBD1* gene expression. Organoids were treated as previously described in Figure S1. Relative mRNA expression levels of *Defa1* **(a)**, *Defa21* **(b)**, *Defa5* **(c)**, *Lyz1* **(d)**, *Reg3γ* **(e)**, and *mBD1* **(f)** were determined by quantitative RT-PCR. Data are presented as means ± SEM (n = 8). Statistical analysis was performed by one-way ANOVA with Dunnett's multiple comparisons test or Kruskal-Wallis test with Dunn's multiple comparisons test. Differences between two groups were analyzed by using unpaired t-test or Mann–Whitney test. Significant differences to PBSO control are indicated as #p-value < 0.05.

Figure S5

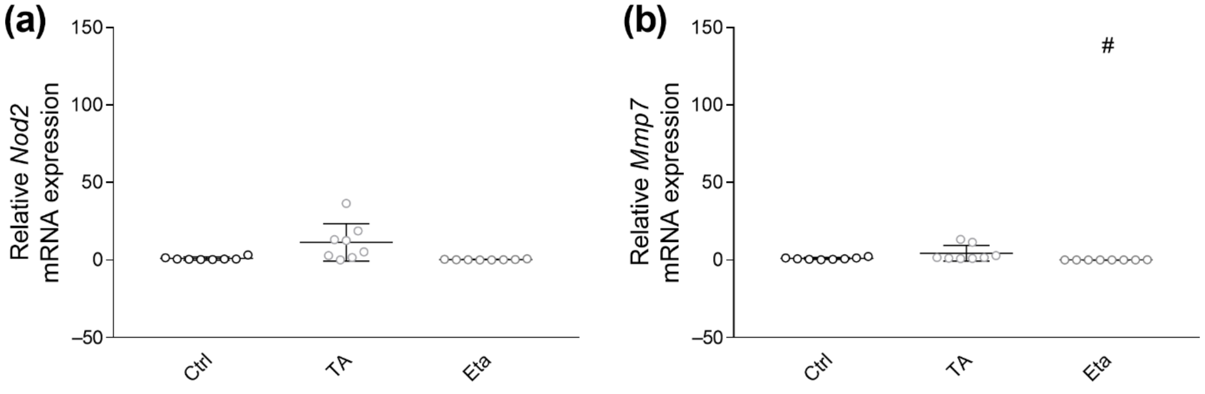

Figure S5. Eta exposure is associated with decreased *Mmp7* gene expression. Organoids were treated as previously described in Figure S1. Relative mRNA expression levels of *Nod2* **(a)** and *Mmp7* **(b)** were determined by quantitative RT-PCR.

Data are presented as means ± SEM (n = 8). Statistical analysis was performed by one-way ANOVA with Dunnett's multiple comparisons test or Kruskal-Wallis test with Dunn's multiple comparisons test. Differences between two groups were analyzed by using unpaired t-test or Mann–Whitney test. Significant differences to PBSO control are indicated as #p-value < 0.05.

Figure S6

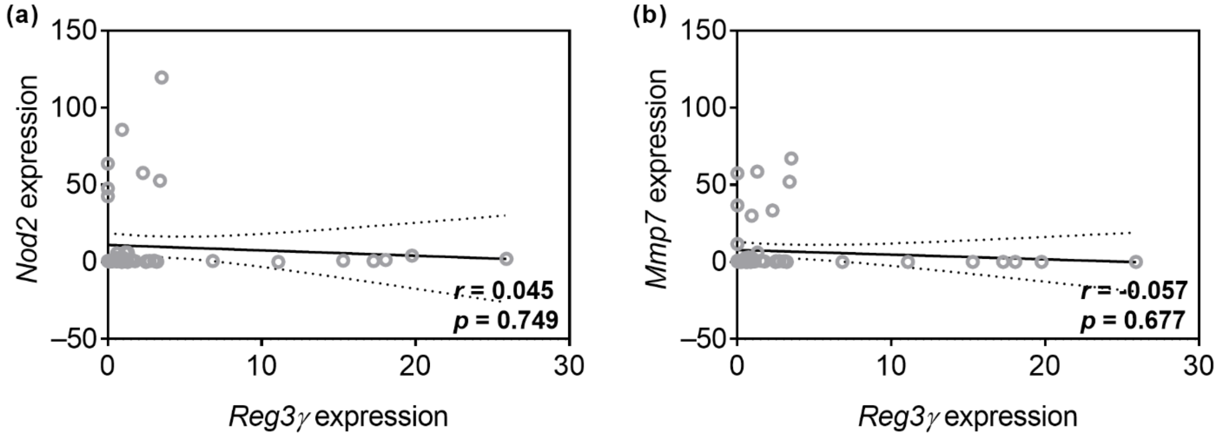

Figure S6. Correlation analysis for *Nod2* **(a)** or *Mmp7* **(b)** and *Reg3γ* mRNA expression. Statistical analysis was performed by two-tailed Spearman rank correlation analysis. Co-efficients in the range of 0.0 to 0.2, or 0.0 to −0.2 were defined as no correlation.
